# The Role of Mu-Opioids for Reward and Threat Processing in Humans: Bridging the Gap from Preclinical to Clinical Opioid Drug Studies

**DOI:** 10.1007/s40429-021-00366-8

**Published:** 2021-04-15

**Authors:** Isabell M. Meier, Marie Eikemo, Siri Leknes

**Affiliations:** 1 Department of Diagnostic Physics, Oslo University Hospital, Sognsvannsveien 20, 0372 Oslo, Norway; 2 Department of Psychology, University of Oslo, Blindern, 0317 Oslo, Norway

**Keywords:** Mu-opioid system, Reward, Stress, Threat, Opioid misuse, Anhedonia

## Abstract

**Purpose of Review:**

Opioid receptors are widely expressed in the human brain. A number of features commonly associated with drug use disorder, such as difficulties in emotional learning, emotion regulation and anhedonia, have been linked to endogenous opioid signalling. Whereas chronic substance use and misuse are thought to alter the function of the mu-opioid system, the specific mechanisms are not well understood. We argue that understanding exogenous and endogenous opioid effects in the healthy human brain is an essential foundation for bridging preclinical and clinical findings related to opioid misuse. Here, we will examine psychopharmacological evidence to outline the role of the mu-opioid receptor (MOR) system in the processing of threat and reward, and discuss how disruption of these processes by chronic opioid use might alter emotional learning and reward responsiveness.

**Recent Findings:**

In healthy people, studies using opioid antagonist drugs indicate that the brain’s endogenous opioids downregulate fear reactivity and upregulate learning from safety. At the same time, endogenous opioids increase the liking of and motivation to engage with high reward value cues. Studies of acute opioid agonist effects indicate that with non-sedative doses, drugs such as morphine and buprenorphine can mimic endogenous opioid effects on liking and wanting. Disruption of endogenous opioid signalling due to prolonged opioid exposure is associated with some degree of anhedonia to non-drug rewards; however, new results leave open the possibility that this is not directly opioid-mediated.

**Summary:**

The available human psychopharmacological evidence indicates that the healthy mu-opioid system contributes to the regulation of reward and threat processing. Overall, endogenous opioids can subtly increase liking and wanting responses to a wide variety of rewards, from sweet tastes to feelings of being connected to close others. For threat-related processing, human evidence suggests that endogenous opioids inhibit fear conditioning and reduce the sensitivity to aversive stimuli, although inconsistencies remain. The size of effects reported in healthy humans are however modest, clearly indicating that MORs play out their role in close concert with other neurotransmitter systems. Relevant candidate systems for future research include dopamine, serotonin and endocannabinoid signalling. Nevertheless, it is possible that endogenous opioid fine-tuning of reward and threat processing, when unbalanced by e.g. opioid misuse, could over time develop into symptoms associated with opioid use disorder, such as anhedonia and depression/anxiety.

## Introduction

**Fig. 1:**
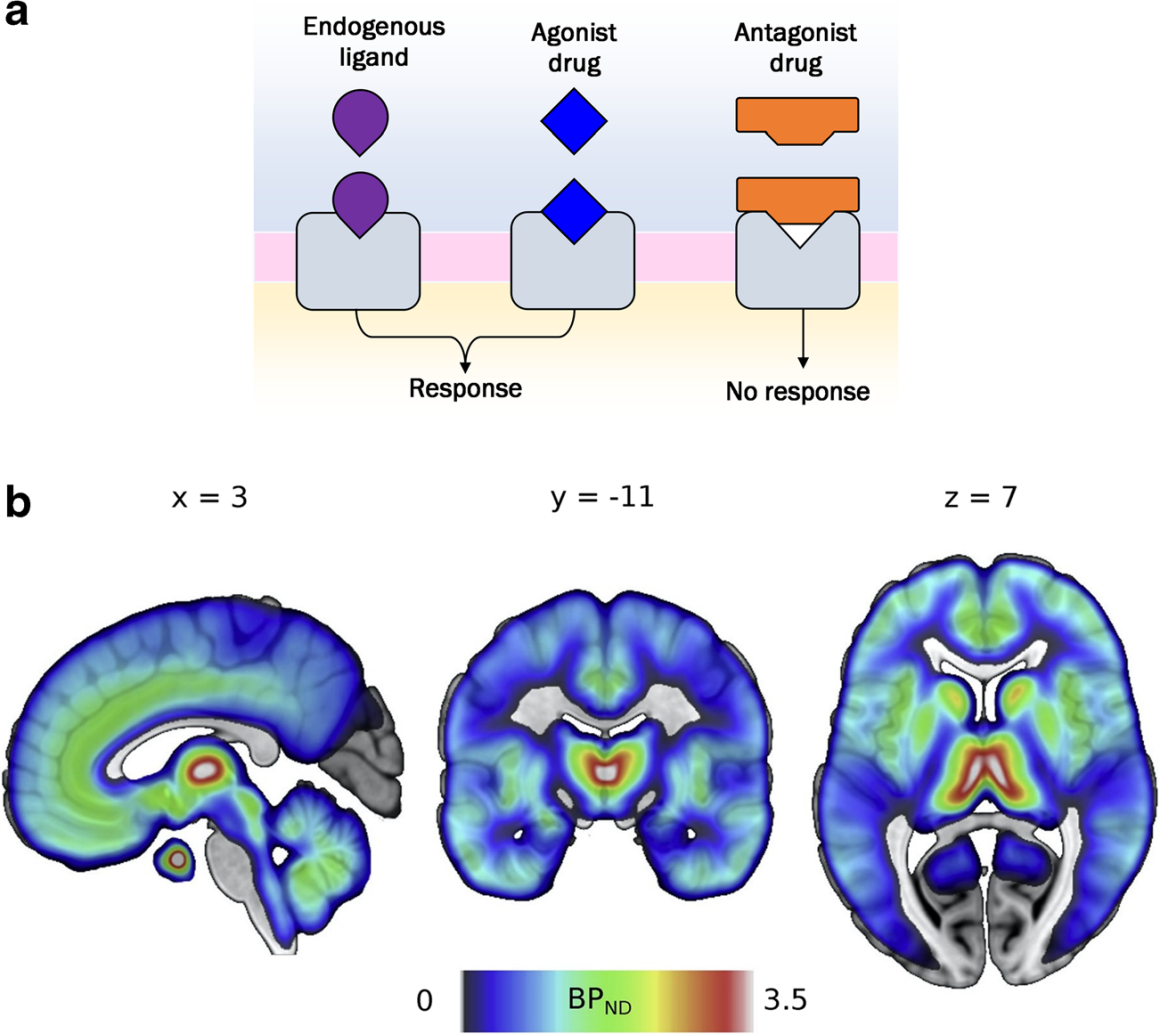
**a** Schematic illustration of the actions of endogenous (agonist) ligands, agonist and antagonist drugs at the receptor. Endogenous ligands and agonist drugs bind to and stimulate receptors. Antagonist drugs bind to and block receptors without stimulating them, thereby preventing other ligands (e.g. endorphins, enkephalins) from activating the receptor. **b** Mu-opioid receptor distribution in the healthy human brain as measured by positron emission tomography using the [11C] carfentanil radioligand BP_ND_ (binding potential relative to nondisplaceable radioligand in tissue, see Innis et al. [58]). Images are based on 204 subjects from Aivo database (http://aivo.utu.fi). Key structures implicated in reward and threat processing are densely innervated with MOR receptors, such as the nucleus accumbens (NAc), the nuclei of the amygdala, the thalamus, anterior cingulate cortex (ACC) and periaqueductal gray (PAG). This MNI-space atlas is available on @VaultNeuro (https://neurovault.org/collections/GCELSAIA/)

Despite increasing awareness of the risks of problematic opioid use, opioid analgesics are still commonly used to treat acute and chronic pain [15, 60, 136]. Opioid analgesics such as morphine and fentanyl primarily bind to mu-opioid receptors (MORs), which are widely expressed in the human brain (Fig. 1b). In addition to pain relief and typical side effects such as nausea and constipation, drugs acting on MORs affect decision making and other cognitive and affective processes (e.g. [122]). Knowledge of the processes modulated by mu-opioid signalling comes from extensive preclinical research as well as experimental and clinical observations of acute and chronic effects of opioid drugs, both agonists and antagonists.

In the literature, the addictive potential of opioids is strongly linked to their ability to induce pleasure and euphoria. Indeed, one of the more robust indicators used to assess the addictive potential of opioid drugs is drug liking [27]. Interestingly, acute subjective responses to opioid administration in healthy, pain-free subjects are highly variable, with many reporting drug disliking [4, 68, 137]. In chronic pain patients receiving opioid treatment, prescription opioid misuse can be driven by a desire for stress relief [76, 79, 102]. Of note, high-stress conditions such as history of trauma, post-traumatic stress disorder (PTSD), low socio-economic status or poor social support are vulnerability factors for development of substance misuse and are frequently observed as comorbidities in opioid use disorder [5, 23, 51, 52, 94, 103, 113, 131]. These findings align with prominent addiction theories pointing towards two major pathways into drug abuse, one related to drug liking and sensitization of the reward circuitry to drug related cues [25, 101], and another more driven by attempts to reduce negative affective states and to cope with stressors [63].

Molecular imaging studies have reported changes in binding potential after chronic exposure to opioid drugs, consistent with altered endogenous opioid function in opioid users [26, 132]. It is unclear whether chronic opioid exposure alters receptor density or endogenous mu-opioid release. For chronic pain, another condition associated with altered mu-opioid binding potential, preclinical work points to changes in receptor density [115]. At the behavioural level, chronic opioid intake has been proposed to reduce responsiveness to natural rewards ([44, 74], but see [36]). Long-term opioid treatment has also been linked to reduced emotion regulation capacity [43], and is highly comorbid with heightened stress sensitivity and negative affect [67, 102]. This leads to the question of whether chronic opioid use could paradoxically reinforce stress, negative affect and anhedonia in the long-term. A similarly paradoxical effect of long-term opioid treatment has been documented for pain (opioid induced hyperalgesia), such that people develop a heightened sensitivity to pain as a result of the opioid therapy [3]. Before we can fully comprehend how chronic opioid use affects behaviour and health, the field needs to establish a thorough understanding of how endogenous opioids modulate behaviour and affect in the healthy human brain. Essentially, to understand what has gone wrong in addiction, we must first establish how these processes function in the healthy, non-addicted human brain.

This narrative review will give an overview of the current state of knowledge on endogenous opioid function in humans, based on behavioural evidence from opioid drug studies. A growing literature of preclinical and human experimental research in healthy subjects has investigated the effects of acute pharmacological administration targeting the mu-opioid receptors. Here we review studies that used drugs to block and/or stimulate the MOR system. Most opioid agonist drugs target mu-receptors (e.g. morphine, hydromorphone). In non-sedative doses, these drugs are thought to inform us about putative functions of endogenous mu-activity. Opioid antagonist drugs (such as naltrexone and naloxone) bind to opioid receptors, thereby preventing endogenous ligands (i.e. endorphins) from binding to and stimulating the receptors (see Fig. 1a). All studies reviewed here employed antagonists at doses thought to yield full MOR blockade (> 90% MOR occupancy at the time of testing). Full blockade studies can test whether an endogenous system is necessary for, or involved in, a behavioural function or experience. The base assumption is that if endogenous ligands are necessary for a behaviour, then that behaviour should be reduced by opioid antagonism.

Specifically, the review will focus on causal evidence on opioid drug effects on reward, stress and threat in healthy people at the behavioural level. Correlational neuroimaging data from PET or fMRI are also discussed where they complement behavioural effects. Each section considers how the human psychopharmacology findings align with results from studies of opioid function in non-human animals. Finally, we also link the knowledge on acute drug effects with findings in chronic opioid exposed groups. For the sake of brevity, opioid regulation of pain falls outside the scope of the present review, which is also limited to the mu-opioid receptor system. Most of the drugs used as antagonists and some of the agonists are non-specific and also bind to kappa and delta receptors; however, doses are optimised for mu-opioid binding. The limited emerging evidence on kappa-specific activity is exciting, see e.g. Darcq & Kieffer [28]; Krystal et al. [65]; Pizzagalli et al. [95], and we note that antagonist effects currently attributed to mu-opioid signalling may also reflect antagonism of kappa and to a lesser extent delta receptors.

## Reward

Both receiving and anticipating rewards can evoke feelings of pleasure in humans. In evolutionary terms, the pleasures of play, food or sex are believed to motivate the individual to engage in these behaviours, securing the individual’s survival and reproductive fitness. Reward processing is often parsed into several components, including liking (the hedonic experience when a reward is anticipated or obtained), wanting (the motivational component, often assessed as the drive and/or effort spent to obtain a reward) and reward-related learning (e.g. [7]).

### Acute Opioid Effects in Presearch

Mu-opioid signalling is tightly linked to liking of palatable foods. Groundbreaking rodent research on the role of the MOR system in reward has identified the areas in the rostrodorsal shell of the nucleus accumbens (NAc), the caudal ventral pallidum (VP), the parabrachial (PB) nucleus as well as the anterior orbitofrontal cortex (OFC) and the posterior insula as hotspots that can enhance ‘liking’ responses to food rewards [8, 9, 19, 92]. Microinjections of a mu-opioid agonist into each hedonic hotspot resulted in increased liking responses to food rewards [8, 19, 92]. Increased liking responses have also been reported from microstimulation in these hotspots using other ligands, including kappa and delta opioid agonists, orexin and compounds binding to endocannabinoid receptors [17–19]. Interestingly, microstimulation with mu-opioid antagonists to block endogenous opioid signalling did not alter baseline ‘liking’ responses in rats [109], although such antagonism has been reported to block hunger-induced increases in food liking [129] and eating [20]. Instead, microstimulation with opioid agonists in so-called hedonic ‘coldspots’ triggers a decrease in food liking responses in rats [92].

Does mu-opioid activation also enhance wanting of food rewards? Rodent studies using striatal microstimulation or systemic drugs indicate a clear role for mu-opioids in food wanting. When rats ate chocolate, striatal enkephalin levels surged [31], and microstimulation of the same area with a mu-opioid agonist resulted in increased palatable food consumption [31]. Opioid agonism in the NAc shell of rodents also increased the motivation of animals to work for food rewards [91, 138], consistent with effects reported after systemic drug administration in rats. Specifically, opioid agonism increases measures of food wanting, whereas antagonism decreases it (e.g., [24]).

Rodent studies also indicate an important role for the MOR system in encoding liking and wanting of non-food rewards such as social play [123]. Social play is an intrinsically rewarding behaviour [116, 124, 125]. Play is thought to be important for social and cognitive development across species and serves a variety of functions including peer-bonding [110, 116, 125]. In rats, play is most frequently observed during adolescence. Infusion of a MOR agonist in the shell and core of the NAc specifically enhanced social play behaviour in adolescent rats [117]. Furthermore, opioid antagonism reduced motivation for social play, the rewarding properties of play and the expression of play behaviour [1]. The opposite pattern was found after morphine. In addition to promoting play behaviours, mu-opioids were also reported to facilitate social novelty seeking in juvenile rats [108]. In voles, mu-opioid signalling in the striatum was found to mediate hedonic aspects of pair bonding [99].

Indeed, neuropharmacological studies across several species point to a role of opioids in mediating reward-related processing. Inhibition of mu-opioid signalling prevented newborn mice [86] and lambs [107] from developing a behavioural preference for their mother, highlighting the importance of opioid signals for social bond formation in these animals. Moreover, a study of five miniature pigs that were given an hour’s access to sweet foods was broadly consistent with rodent work, indicating putative reward-related opioid release in the pig NAc [133]. Whereas in cats, opioid antagonism reduced cats’ engagement with catnip-like substances, putatively due to blockade of its intrinsic reward value [120]. Further to these reports across mammals, opioids are even reported to mediate certain types of singing in songbirds. For instance, fentanyl injections increased non-communicative, intrinsically rewarding singing in starlings [112].

### Acute Opioid Effects in Human Research

Overall, animal research indicates that opioid signalling impacts a large range of potentially rewarding behaviours such as feeding, bonding and play. Does the human opioid system exhibit similar modulation of reward processing? Due to methodological limitations, it is as yet unknown whether the human brain contains anything like the mu-opioid sensitive set of hedonic hotspots capable of enhancing food liking responses in rodents. PET imaging indicate rich expression of MORs in the human striatum, as well as in the thalamus, insulae, amygdala and anterior cingulate cortex (Fig. 1b). Overall, however, the available evidence from systemic drug studies in healthy humans is consistent with the rodent evidence that MOR signalling promotes liking and wanting of high-calorie foods. A study from our lab indicated modest increases in sucrose liking after treatment with a low-dose mu-opioid agonist [37]. There is also consistent evidence that opioid blockade decreases reports of food enjoyment and reduces high caloric food consumption in humans [37, 134, 135, 139]. A recent study also reported decreased effort to obtain food reward after MOR antagonism, but no effect on subjective ratings of food liking or wanting [64].

**Fig. 2:**
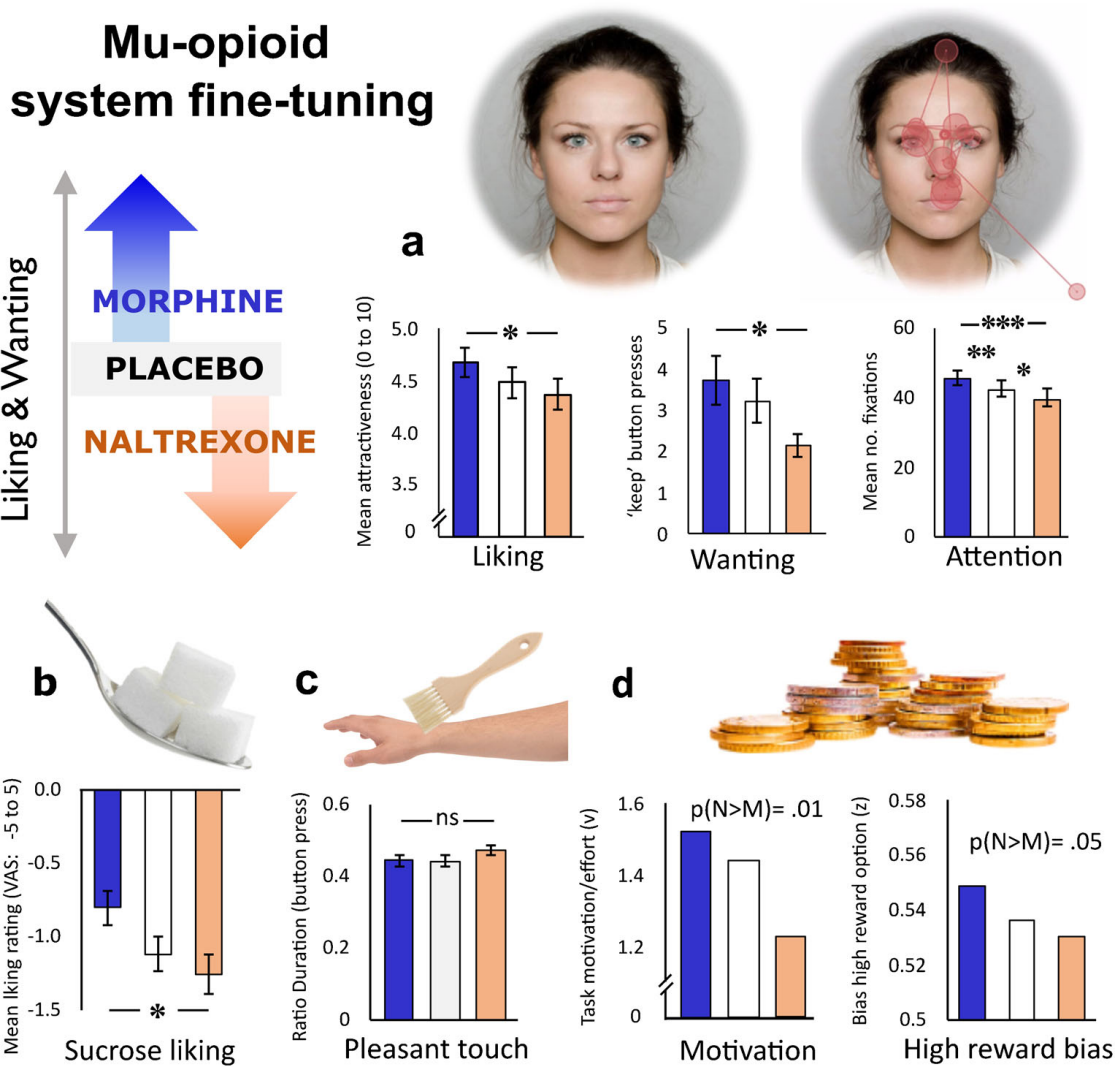
Overview of behavioural results from our lab’s investigation into the role of endogenous mu-opioid signalling for reward liking and wanting in healthy young men. We reasoned that behaviours increased by 10 mg per-oral morphine and decreased by 50 mg of the non-specific opioid antagonist naltrexone, would be behaviours likely to be promoted by endogenous mu-opioid signalling in the healthy human brain. In this repeated-measures pharmacological administration study, the expected pattern of results was found for most reward domains, with the exception of gentle caress-like touch. Notably, these bidirectional drug effects are unlikely to result from mood and/or side effects of the drugs; after the three sessions, participants remained fully blinded to the drug order (chance level guesses). **a** Faces: liking ratings most attractive opposite-sex faces; effort exerted to see most attractive faces [21]; visual exploration and attention (fixations) to others’ eyes as measured with eye-tracking [22]. Gaze pattern illustrates a single trial. **b** Sweet liking ratings of high sucrose drinks [37]. **c** Ratio of time spent on most comfortable brush speed [70]. **d** Monetary reward: parameters indicating response bias for a high reward stimulus (shift in decision starting point, z) and total effort exerted (motivation; drift rate, v) from a Bayesian drift diffusion model [35]. * denotes p < 0.05, ** < .01,*** p<0.001, where frequentist statistics were used (**a**–**c**). P(M>N) denotes the posterior probability of the contrast - that the decision parameter estimate of naltrexone is greater than morphine (N>M)

In line with rodent work showing opioid modulation specifically of high reward options in the food and social domains, several human studies targeting mu-opioid receptors with systemic agonists and/or antagonists report drug modulation of high-value reward responses. As illustrated in Fig. 2, our lab conducted a randomised, double-blind, placebo-controlled study in which healthy young men were treated with naltrexone or morphine before exposure to social, food and abstract rewards. We found the expected bidirectional modulation of responses to high-calorie foods [37], attractiveness judgements and viewing time of beautiful photographed faces [21], and for computationally derived measures of reward preference and motivation for high-value monetary gains [35]. We also found that morphine increased and naltrexone decreased visual attention to the eyes of human faces [22], consistent with the interpretation that the human endogenous MOR system promotes attention to socially relevant cues and thereby facilitates detection of reward cues. This, in turn, could influence reward-related learning and memory. In line with this idea, Syal et al. [114] reported MOR agonist effects on reward-related learning and memory, with increased recall of social reward cues (happy faces) in an object relocation task [114].

Results from other labs are broadly consistent with the above pattern of results. Buchel et al. [14] used fMRI together with MOR blockade and reported decreased pleasure ratings when young men viewed erotic photographs and cues of monetary reward, as well as reduced reward-related activation to erotic stimuli in the ventral striatum, amygdala, hippocampus, orbitofrontal cortex and medial prefrontal cortex. Petrovic et al. [93] similarly reported larger effects of naloxone for high-value reward outcomes, which correlated with activity reductions in the rostral anterior cingulate. Administration of naltrexone also reduced motivation to exert effort for chocolate rewards in a pavlovian instrumental transfer task [130], attenuated motivational ratings following almost-wins in a gambling task [96], diminished learning in a reward-driven reinforcement learning task [34] and decreased physical effort to obtain a reward [64]. Some of these studies, including our own, tested only men due to what we now know is a misguided belief that female hormonal fluctuations would add more noise than those of males [106]. However where women have been tested, results are broadly comparable to studies of men only.

Korb et al. [64] also reported opioid antagonist effects on facial muscle activity (more frowning during reward anticipation; less smiling during consumption of food rewards, in the absence of changes in subjective valuation of these rewards [64]). Increased negative facial reactions after opioid blockade were also reported during viewing of smiling faces [84], consistent with a role for MOR signalling in automatic behavioural responses (mimicry) that support social bonding [66]. Moreover, opioid antagonism has been reported to reduce ratings of the *feeling* of being connected to other people [56, 57]. The magnitude of the shift in feelings of connectedness is modest, comparable to effect sizes of acute opioid drug effects on other high-value rewards. The reviewed literature on acute opioid drug effects broadly point towards human endogenous mu-opioids promoting approach of high-value rewards in humans, consistent with findings in non-human animals. However, there are also several discrepancies in the literature. Whilst most published findings are consistent with opioids promoting appreciation and approach of rewards [122], at least three studies have reported comparable or even more enjoyment of caress-like touch after opioid blockade ([16, 64, 70] - see Fig. 2c). Differences in reported drug effects could be related to distinct processing of reward types (magnitude/type) as well as other study design choices. Considering the relatively modest effect sizes and typical sample sizes ranging from 10 to 50 healthy participants in these psychopharmacological studies, statistical power is also a concern. More research is needed to provide a full understanding of the functions of the healthy opioid system.

Importantly, the human literature using antagonists unequivocally demonstrates that a substantial amount of reward liking can be observed (see Fig. 2) even after so-called opioid blockade, defined as 90–100% of MOR bound by naloxone/naltrexone or other antagonists. It is also pertinent in this context to recall that whilst several PET studies have yielded results consistent with release of endogenous mu-opioids during and/or after engagement in rewards such as social acceptance [53], eating pizza [119] or enjoying comedy TV [75], other studies have yielded opposite findings, e.g. for pleasant touch [89]. In other words, it is clear that whilst endogenous mu-opioids contribute to the fine-tuning of reward processing in healthy humans, mu-opioid receptors are not the only neurochemical system capable of signalling reward liking and motivation in the human brain. Other important modulators and mediators of reward processing in humans include endocannabinoids, serotonin and dopamine [11, 39, 78, 105]. In the next section, we discuss the role of opioids for negatively valenced events, stress and fear.

## Stress and Fear

Being able to adequately respond to threat cues in the environment is crucial for human social functioning. Hypersensitivity to threat and impaired threat-related learning processes are associated with affective disorders such as anxiety or PTSD [49, 98]. Rodent and human experimental research indicates a regulative and supportive role of MOR signalling in adaptive responses to threat [38, 48, 80]. As illustrated in Fig. 1, key brain structures involved in the processing of threat are densely innervated with mu-opioid receptors: the nuclei of the amygdala, the thalamus, anterior cingulate cortex (ACC) and periaqueductal gray (PAG) [82, 97].

### Acute Opioid Effects in Preclinical Research

There is abundant evidence of MOR modulation of threat-related learning in rodents. Endogenous mu-opioid signalling inhibits fear conditioning [46] and facilitates fear extinction [82]. Moreover, administration of a MOR agonist interfered with the aversive conditioning process, decreasing its efficacy, whereas administration of the MOR antagonist naloxone enhanced threat conditioning in rodents [80, 83]. The peri-acqueductal gray (PAG) emerges as a key structure mediating opioid regulation of threat-related learning. Opioid receptors in the ventrolateral part of the PAG (vlPAG) are suggested to mediate discrepancies between an actual and an expected outcome of a conditioning trial which is known as the prediction error and therewith influence both, the acquisition of conditioned fear responses as well as extinction learning [80, 82]. For example, when outcomes are less aversive than expected, opioid signalling from the vlPAG accompanies these (relative) safety cues, triggering a downregulation of threat responses in the amygdala and thereby reducing the discrepancy between expected and experienced outcomes, facilitating extinction learning [80]. A recent study found that MORs in the dorsal midline thalamus contribute to opioid modulation of extinction learning [6] and effects in the same direction were found in a study using systemic blockade of the MOR system [62].

The MOR system also regulates stress responses. Early infant-attachment studies in different mammalian species showed that opioid blockade increased distress vocalisations in young animals after separation from their mothers (for a review, see e.g. [71]). Administration of MOR agonists on the other hand, resulted in a decrease of distress vocalisations [90]. Human and non-human animal research reports that endogenous opioids are released in response to stress and are directly involved in regulating the HPA axis response to stressors [2, 30, 88, 104, 126, 127] in a sex-specific manner. Rodent research investigating the interactive role of the MOR system and the corticotropin-releasing factor (CRF) in the locus coeruleus, shows that at confrontation with an acute stressor the MOR system is involved in downregulating the effects of CRF, promoting recovery after stress. This mechanism is highly adaptive in the short-term; however, confrontation with repeated stress resulted in chronic inhibitory action via mu-opioid receptors in the locus coeruleus, with long-term modifications of neural circuits involved in stress regulation [121].

### Acute Opioid Effects in Human Research

In line with rodent evidence [46, 81], two psychopharmacological neuroimaging studies demonstrated an inhibitory role for the MOR system in the acquisition of conditioned fear in humans [38, 48]. Blocking the MOR system with naloxone resulted in a modest increase in behavioural conditioned responses, defined as faster identification of the stimulus location of conditioned stimuli, and reduced habituation to conditioned stimuli in pain- and threat-related pathways [38]. Furthermore, administration of naltrexone was associated with a lack of habituation response to threat cues in the amygdala over time. More recently, these findings were extended to the social learning context. Haaker et al. [48] showed that conditioned responses acquired through observational learning were enhanced after blocking the MOR system with naltrexone as indicated by increased signalling responses in amygdala, PAG and midline thalamus. Behaviourally, MOR blockade also led to stronger long-term expression of learned threat responses [48]. Together, the results support the idea that endogenous opioid signalling dampens aversive learning processes through first-hand experience as well as through social learning.

Does human MOR signalling regulate sensitivity to negative affective cues outside of a fear learning context? There is some evidence in support of this notion from behavioural studies. Administration of a MOR agonist reduced sensitivity to fearful facial expressions in an emotion recognition task [59], whereas Løseth et al. [69] reported reduced perceived anger in images with neutral and ambiguous facial expressions only. In another study, administration of buprenorphine resulted in an initial attention bias to fearful emotional expressions, as measured by direction of gaze with electrooculography [13]. Notably, the effects in these studies were small and somewhat inconsistent, pointing to a relatively minor role of mu-opioid activation in perception of emotional expressions. Beyond emotion perception, blockade of the MOR system resulted in a stronger negative subjective experience of losing money in a gambling task in an fMRI set-up. Experiencing losses was associated with enhanced activity in the anterior insula and the caudal anterior cingulate cortex [93]. Overall, there is some support of the idea that endogenous opioid activity in healthy humans reduces the sensitivity to negative affective cues ([13, 59, 69, 93]; but see [128]), but effects tend to be small.

In addition to mu-opioid fine-tuning of threat-related learning and perception of negative affective cues, endogenous opioid signalling also impacts the body’s stress response via its actions on the hypothalamic–pituitary–adrenal (HPA) axis [2, 72, 126]. These effects are sex-dependent. For instance, opioid antagonism has revealed a substantial tonic mu-opioid inhibition of cortisol and ACTH that appears to be specific to women [72].

The role of MOR for (hypothetical) social rejection was tested using PET: the bilateral amygdala, the ventral striatum, thalamus and the subgenual anterior cingulate cortex showed decreased binding after rejection [53]. As has also been reported for physical pain, e.g. [141], MOR activity (or alternatively a change in mu-opioid receptor expression) was positively correlated with measures of successful downregulation of negative affect [53]. Nevertheless, the role of MOR signalling in regulating the human subjective experience of distress may be substantially smaller than its effect on physiological stress responses. Administration of the MOR agonists compared to placebo reduced the cortisol stress-response in humans [10, 12], as reported in abundant rodent studies [33, 100, 121]. Unexpectedly, this dampening of cortisol was not associated with opioid-induced decreases of subjective ratings of anxiety or negative emotions [10, 12]. Conversely, opioid antagonism increased cortisol after stress induction without altering subjective stress responses [2].

Overall, the evidence of acute opioid effects on stress and threat processing in healthy humans suggests a modest, protective function of endogenous opioid signalling—reducing the physiological stress response and fine-tuning sensitivity as well as learned responses to negative stimuli. Whether chronic dysregulation of endogenous opioid signalling, through e.g. chronic opioid drug use, could paradoxically reinforce stress responses and negative affect is of yet unknown. Current evidence indicates co-occurrence of opioid use disorder and anxiety-related symptoms [67, 76, 79, 102], (history of) life stressors such as trauma or low socio-economic status [51] and poor emotion regulation capacities [43]. More systematic research of the healthy human brain, addressing the role of the MOR system in fear-related learning and other negatively valenced processes, is necessary to understand how regulation of negative affect or learning processes could be altered in individuals with chronic opioid use.

### Anhedonia and Reward Sensitivity Following Prolonged Opioid Use

Disrupted reward processing related to drug-induced disturbances of the mu-opioid (and dopamine) reward system is a key element in neurobiological theories of drug addiction (e.g. [55, 63, 101]). Whilst the effects of acute administration of opioids on reward sensitivity in healthy participants are broadly consistent, the evidence for anhedonia following long-term opioid use and dependence is mixed [41, 50, 61].

The knowledge of anhedonia in chronic opioid use and opioid use disorder is largely based on individuals with an established opioid use disorder. Consequently, disentangling opioid-induced changes in reward behaviour and experience from pre-existing anhedonia is challenging. Additionally, opioid use disorder is associated with a range of psychosocial vulnerability factors [29] that in themselves increase risk of anhedonia and blunted reward responsiveness. For example, comorbid mental health symptoms and disorders (e.g. [47]); stressful life events, (history of) trauma and even PTSD (e.g. [85]); unstable social relations and economic resources [32] and somatic disorder such as chronic pain [42] may independently impact reward experience and behaviour. Furthermore, the existing literature typically reports data from polydrug users who regularly use nicotine and sometimes have high alcohol consumption and use other psychotropic medication (e.g. anxiolytics, hypnotics). As substance use disorder by definition is a chronic relapsing disorder, the patients tested during medication-assisted treatment (MAT) with opioid agonist drugs are rarely stable in treatment, and often consume illegal drugs on-top of treatment.

A number of studies observe blunted sensitivity to natural rewards in, or following, long-term opioid use as measured by behavioural tests and/or brain imaging and higher self-reported anhedonia [40, 44, 54, 73, 77, 111, 140] compared to control participants. Other studies report little change in reward experience and behaviour following long-term opioid use. For instance, we have reported intact objective and subjective reward responsiveness in women in stable long-term MAT with buprenorphine or methadone relative to healthy volunteers [36]. We have also found that self-reported anhedonia was not elevated in chronic pain patients treated with opioids, compared to patients who did not use opioids [45]. Pain patients who specifically reported misusing their opioid analgesics however, showed higher levels than non-misusing patients. Notably, these results did not change when adjusting for variance in depression symptoms [45]. Moreover, in the domain of sweet taste reward, long-term opioid use has been associated with *increased* sucrose liking, motivation and consumption [142], indicating a lack of anhedonia for sweet foods in this population. However, this apparent increase in reward responsivity may be caused by a blunted sweet taste reward experience and need for higher sucrose content to reach ‘optimal’ sweetness [143].

Several reviews on anhedonia in substance use disorders show that the most convincing evidence for anhedonia in OUD and other SUDs is related to discomfort during early abstinence in patients who have started treatment, and correlates with drug craving and opioid use [41, 50, 61]. In a recent study, Garfield et al. [40] found evidence to suggest that illicit opiate use is a potential cause of self-reported anhedonia among patients in MAT with opioid agonist drugs. Across samples reported in the literature, we find broadly comparable anhedonia scores in chronic pain and substance use disorder [118]; it is conceivable that anhedonia in these conditions is more related to discomfort and suffering than to direct effects on opioid receptors in the reward system.

In a large-scale systematic review and meta-analysis previously published by our group [118], we also show that the degree of anhedonia reported in substance use disorder is typically much smaller than that measured in for example major depressive disorder or PTSD. The relatively intact responsiveness to non-drug reward exhibited across studies of substance use disorder samples is somewhat unexpected, considering the adversities and risk factors associated with OUD. Overall, we conclude that there is little evidence to suggest that the anhedonia and reward changes are due to opioid-specific effects. Instead, anhedonia in OUD may be explained by other co-occurring factors. An account of how opioid gene variability is related to OUD is reviewed in Darcq & Kieffer, [28] and Moningka et al. [87]. Notably, the number of participants in most studies is modest due to the participant group being notoriously hard to recruit, test and follow over time. The very heterogenous samples give the studies ecological validity, but prevents researchers from determining the unique role of opioid agonist exposure on reward responsiveness. We believe that multicentre studies may be needed to increase the quality of evidence of anhedonia related to chronic opioid exposure.

## Conclusion

The available human psychopharmacological evidence clearly points to MOR system fine-tuning of reward and threat processing in the healthy brain. Mu-opioid signalling can moderately increase responses of liking and wanting to a range of reward modalities, including foods, social information, abstract rewards and social connectedness. Whilst most studies report significant effects to especially high-reward stimuli, inconsistencies with regard to reward type (e.g. touch) remain. With regard to threat processing human evidence points towards an inhibitory role of the MOR system in the acquisition of fear associations. However, whilst the direction of the effects is consistent with findings in rodents, the magnitude of effects is not comparable to those reported in rodent work. Results from perception studies are mixed but suggest a subtle reduction of sensitivity to negative affective stimuli via endogenous opioid signalling. Similarly, human studies investigating the regulatory role of the MOR system in stress responses indicate subtle effects on the subjective experience of stress, but large effects on the physiological level. We venture that the role of endogenous mu-opioids for human reward and threat is limited to fine-tuning the responses, and that the human brain also draws on other neurotransmitter systems such as the dopamine, serotonin and endocannabinoid systems in reward and threat-related processing.

More research is warranted to gain a better understanding of opioid modulation of reward, threat and stress-related processing in the healthy human brain as well as in opioid users. Despite high co-occurrence of opioid misuse and anxiety, it is unclear whether anxiety predates or could result from chronic opioid exposure. Anhedonia is often, but not always observed in opioid using populations, but changes in reward sensitivity observed after chronic opioid use may be explained by other co-occurring vulnerability factors rather than opioid system dysregulation.
